# Moving beyond Definitive Therapy: Increasing Physical Activity in Survivors of Cancers of the Head and Neck

**DOI:** 10.3390/curroncol29020103

**Published:** 2022-02-17

**Authors:** Anthony D. Nehlsen, Kunal K. Sindhu, Brianna M. Jones, Eric J. Lehrer, Jared P. Rowley, Richard L. Bakst

**Affiliations:** Department of Radiation Oncology, Icahn School of Medicine at Mount Sinai, New York, NY 10029, USA; anthony.nehlsen@mountsinai.org (A.D.N.); brianna.jones@mountsinai.org (B.M.J.); eric.lehrer@mountsinai.org (E.J.L.); jared.rowley@mountsinai.org (J.P.R.); richard.bakst@mountsinai.org (R.L.B.)

**Keywords:** exercise, head and neck cancers, physical activity, pre- and re-habilitation, survivorship

## Abstract

As chemotherapeutic, radiation, and surgical techniques have improved, there has been a dramatic improvement in survival in patients diagnosed with cancers of the head and neck. As a result, a heightened focus on survivorship by clinicians will increasingly prove necessary. In particular, medical care teams will have to pay special attention to mitigating the long-term sequelae of definitive cancer treatments, many of which act as barriers to exercise. This is unfortunate, as the benefits of exercise in patients with cancer have become increasingly recognized. In this review, we discuss the potential benefits of and barriers to exercise in survivors of cancers of the head and neck. We also review existing exercise guidelines and strategies by which clinicians can promote exercise in this unique patient population.

## 1. Introduction

Cancers of the head and neck account for approximately 54,000 new cases, representing 3% of all new cancer diagnoses, and 11,000 deaths per year in the United States [1]. As chemotherapeutic, radiation, and surgical techniques have improved in recent years, there has been a dramatic improvement in survival rates in patients with cancers of the head and neck. According to the SEER database, the five-year relative survival for such patients was approximately 66.7% from 2011 to 2017 [2,3]. However, the definitive treatments for cancers of the head and neck, which are employed with curative intent and include surgery, radiation alone, and chemoradiation, can cause a plethora of treatment-related side effects. An analysis of the ECOG-Acrin 2399 trial, for example, found that patients had a significant decline in quality of life, swallowing, and speech function following chemoradiation [4]. Regrettably, only a limited base of cancer survivorship literature exists that examines this cohort of patients.

The terms “physical activity” and “exercise” are often used interchangeably in the medical literature. However, it is important to understand the differences between the two. While physical activity can be defined as any bodily movement that results in the use of energy, exercise describes physical activity that is purposeful and structured with the goal of improved physical fitness [5]. Exercise encompasses both aerobic exercise, which aims to improve cardiovascular fitness, and resistance or anaerobic exercise, which aims to improve muscular strength [6].

The benefits of exercise in patients with cancer have become increasingly recognized. Several studies have demonstrated improvements in functional, quality of life, physical, and survival outcomes with structured exercise regimens in patients with cancer [7,8,9]. A recent meta-analysis by Burgos-Mansilla et al., for example, found that exercise interventions were associated with a trend towards improved quality of life and low rates of adverse effects [10]. However, patients with cancers of the head and neck represent a minority of the patients included in many studies evaluating the utility of exercise interventions, and a myriad of treatment- and disease-related side effects often limits their ability to engage in exercise programs. Midgley and colleagues, for example, found that dry mouth or throat, fatigue, shortness of breath, muscle weakness, dysphagia, and shoulder weakness and pain were the most common barriers to exercise in patients with cancers of the head and neck [11]. Several studies have also found a high prevalence of major depression in this cohort of patients, and a review by Barber et al. found that depression in patients with cancers of the head and neck was an independent predictor of inferior survival [12,13,14]. Given these findings, oncologists must devote greater attention to quality of life and psychosocial distress in patients with cancers of the head and neck prior to, during, and following the completion of definitive treatment. Additionally, these patients may also have other contraindications to exercise interventions, including extended periods of postoperative recovery, severe malnutrition, pain, anemia, and/or significant cardiopulmonary comorbidities. In patients with cancers of other anatomic sites, such as of the breast and prostate, both aerobic and resistance exercises have been shown to alleviate many treatment-related adverse effects, such as cancer-related fatigue, cognitive decline, physical functioning, and psychiatric and emotional disturbances. Thus, oncologists must devote greater attention to ensuring that patients with cancers of the head and neck complete sufficient exercise before, during, and following the completion of definitive treatment.

National guidelines recommend that patients with cancer meet the public health guidelines for exercise. The American College of Sports Medicine (ACSM) has published guidelines for fitness testing and exercise programs for patients with a variety of malignancies [15]. Overall, however, there is a lack of specific guidance for patients with cancers of the head and neck regarding the intensity and structure of exercise regimens. This is unfortunate, as prior studies have noted that certain exercise interventions have resulted in improvements in several cancer-related endpoints, including anxiety, body mass index, bone density, depression, and fatigue [16,17,18].

Given the lack of specific guidance, it is perhaps unsurprising that there has not been an increase in structured exercise programs to improve clinical, functional, and psychological outcomes in survivors of cancers of the head and neck. Even as they experience better survival outcomes with modern therapy techniques, these individuals continue to face a multitude of barriers following surgery, chemotherapy, and radiotherapy treatments that prevent full participation in exercise. In this review, we will discuss the importance of and barriers to exercise in patients with and survivors of cancers of the head and neck, the state of current exercise guidelines for patients with cancer, and interventions to improve participation in exercise by survivors of cancers of the head and neck.

## 2. The Importance of Exercise in Survivors of Cancers of the Head and Neck

In recent years, an increasing number of studies have concluded that exercise before, during, and after oncologic treatment has broad beneficial effects in patients with cancer [19]. A 2009 review of nearly 150 studies on the topic, for example, concluded that exercise “has beneficial effects on a wide variety of physical fitness and [quality of life] endpoints in cancer survivors including functional capacity, muscular strength, body weight and composition, flexibility, fatigue, nausea, diarrhea, pain, physical well-being, functional well being, depression, anxiety, rigor, anger, mood, self esteem, satisfaction with life and overall quality of life”. Moreover, the authors noted that the examined studies suggested that exercise might reduce the risk of or slow cancer progression [20]. Similarly, a 2017 systematic review found that physical activity before, during, and after oncologic treatment was linked to decreased fatigue, improved physical functioning, and higher quality-of-life in patients with cancer [9].

Additionally, a 2019 systematic review and meta-analysis, which examined evidence from 136 studies, found that higher levels of exercise both pre- and post-diagnosis were associated with improved overall survival in patients with at least 11 types of cancer [7]. A more recent review of the literature, from 2021, noted that exercise programs “mitigate many of the complications and risks associated with cancer, particularly thromboembolism, fatigue, weight gain, arthralgia, cognitive impairment and depression” [21].

Moreover, exercise appears to be safe and well-tolerated in patients undergoing radiation therapy. A recent systematic review of 1536 patients treated across 26 different studies noted improvements in multiple patient-reported outcomes including quality of life, anxiety, fatigue, and mood in patients undergoing combined exercise and radiation therapy. However, it should be noted that of the studies included, only 2.8% comprised patients with cancers of the head and neck [22]. Another systematic review and meta-analysis of six randomized control trials of patients with prostate cancer who received radiation therapy published in 2021 by Schumacher et al. noted statistically significant improvements in cardiovascular fitness, muscle function, and rates of urinary toxicity in men who exercised [23]. Thus, while there are limited data available in the head and neck cancer population, multiple studies have demonstrated significant improvements in patient-reported outcomes across multiple malignancies.

Structured exercise programs may be of particular benefit to patients with cancers of the head and neck. In a randomized controlled trial published in 2019, 148 patients with cancers of the head and neck undergoing definitive chemoradiation received either an eleven-week structured exercise program consisting of both aerobic and resistance exercises or standard physical activity recommendations without supervision. The patients who participated in the structured exercise program experienced significant improvements in cancer-related fatigue, functional capacity, and quality of life as compared to the patients who received standard physical activity recommendations without supervision [24]. Given all of these benefits, increasing the amount of physical activity performed by patients with and survivors of cancers of the head and neck is crucial.

## 3. The Barriers to Exercise in Survivors of Cancers of the Head and Neck

Previous studies of adults have identified numerous barriers to exercise among individuals in the general population. Commonly cited barriers include a lack of time, access to facilities and equipment, and exercise partner. Additionally, individuals who do not carry out sufficient exercise often cite the poor quality of their neighborhoods and local weather, which can influence the availability and feasibility of outdoor exercise [25,26]. Among older adults, physical limitations, often tied to chronic health conditions, are another important factor [27,28].

The COVID-19 pandemic has also posed special challenges to individuals seeking to exercise. A recent survey from Brazil of 1570 respondents, for example, found that “laziness and fatigue”, a “lack of motivation”, a “lack of appropriate equipment/facilities/space”, and a “lack of time” were the most commonly identified barriers to physical activity during the COVID-19 pandemic [29]. Another recent survey of 1,361 cancer survivors found that 32% of patients exercised less during the first six months of the pandemic. Patients who were unemployed or retired, undergoing active treatment, had increased alcohol consumption, or were managing additional psychosocial stressors were among those more likely to experience a decrease in their activity level [30]. As the COVID-19 pandemic continues, and with the threat of disease outbreaks in the future, this information is crucial for oncologists to keep in mind when recommending and designing exercise regimens for their patients [31,32].

Individuals diagnosed with cancers of the head and neck are subject to the same barriers when it comes to obtaining sufficient exercise. However, they also face special challenges. Nearly half of patients diagnosed with cancers of the head and neck each year are 65 years of age and above [33]. Moreover, the lifestyle factors that play a role in the development of many cancers of the head and neck, including alcohol and tobacco use, are also involved in the pathogenesis of serious comorbidities, such as cardiac and pulmonary disease, that may impede patients from regularly exercising [34].

**Figure 1:**
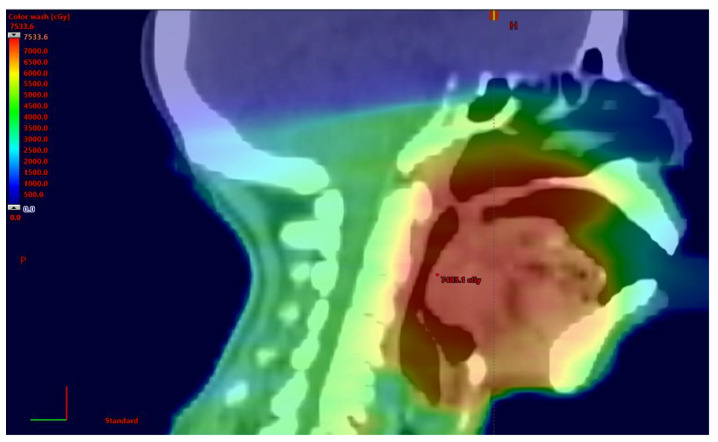
Depiction of a radiation therapy plan for a patient who was treated with a dose of 70 Gray for a squamous cell carcinoma of the right tonsil. Note the significant radiation doses delivered to the surrounding normal tissues.

Additionally, the symptoms associated with the development and treatment of cancers of the head and neck, including dysphagia, dysgeusia, fatigue, xerostomia, and weight loss, can interfere with the ability of patients to consistently adhere to rigorous exercise programs. Patients receiving radiation therapy, for example, receive significant doses of radiation to their normal tissues, including their oral cavities, pharyngeal constrictors, and salivary glands, which can impact the function of these tissues after the completion of treatment (Figure 1). Damage to the major and minor salivary glands, for example, may lead to the permanent loss of taste and persistent xerostomia, while fibrosis of the tongue and pharyngeal constrictors may result in persistent dysphagia and odynophagia. Both of these long-term side effects can make it difficult for patients to maintain their weight. In addition, significant fractions of survivors of cancers of the head and neck experience persistent fatigue, shortness of breath, and weakness after treatment, all of which compromise their ability to exercise. These serious treatment-related sequelae can act as further deterrents to exercising regularly [11].

Psychological distress can also play a significant role. As previously mentioned, the prevalence of mental health disorders, including anxiety-related disorders and depression, is significantly higher among patients with cancers of the head and neck than the general population [35]. Prior studies have found that low mood and stress act as significant barriers to exercise among individuals with these conditions [36]. Moreover, concerns about body image and low self-esteem, both of which are not uncommon among patients with and survivors of cancers of the head and neck, further act as obstacles to exercise among this population [37,38].

As a result, few patients with cancers of the head and neck obtain sufficient exercise, either prior to or after their cancer diagnoses. A 2006 study by Rogers et al., for example, found that just 30.5% of patients with cancers of the head and neck at a single academic institution met physical activity guidelines for exercise prior to their cancer diagnoses. Despite exercise’s positive associations with emotional, functional, physical, and social well-being, this figure dropped to just 8.5% after their diagnoses [39].

Identifying methods to combat many of these barriers to exercise will be important in improving adherence with exercise prescriptions in the future. One such method is encouraging home-based exercise regimens that are simple and require limited equipment. A recent systematic review by Batalik and colleagues demonstrated that home-based interventions were generally effective in improving quality of life, cardiorespiratory fitness, levels of fatigue, and body composition [40]. The studies examined noted a high rate of treatment compliance, with only one study reporting an adherence rate of less than 71%. Finally, although safety data were relatively scant, adverse effects were generally limited to musculoskeletal events. These data suggest that home-based interventions may be an effective option, particularly for patients with limited time, resources, or other barriers to exercise.

## 4. Current Guidelines

As noted above, promoting exercise represents a safe and effective intervention for improving outcomes in patients carrying a cancer diagnosis. However, defining a generalized and practical prescription for exercise in this population remains challenging due to a number of complicating factors, including comorbid conditions and adverse effects from previous cancer treatments, which can vary widely among individual patients [9]. To date, no specific exercise guidelines exist for survivors of cancers of the head and neck. However, multiple broader efforts have been made in recent years to provide exercise guidance to specific patient populations.

In 2008, the United States Department of Health and Human Services (HHS) developed their Physical Activity Guidelines for Americans (PAGA), which recommended completing at least 150 min of moderate-intensity or 75 min of vigorous-intensity aerobic exercise per week; resistance training exercises targeting the major muscle groups at least twice per week; and flexibility exercises at least twice per week [41]. However, they did not make any specific recommendations regarding how patients who had or survived cancer should approach exercise or how much activity they should aim to complete. To address these shortcomings, the ACSM developed exercise guidelines for cancer survivors in 2010 [15]. These guidelines recommended that survivors of breast, prostate, colon, gynecologic, or hematologic cancers follow the PAGA guidelines for aerobic exercise, while those who had undergone stem cell transplants begin with less intensive exercise regimens. Furthermore, with the exception of breast cancer patients, who were cautioned to begin with low-intensity resistance training to reduce arm and shoulder symptoms, all other patients were instructed to follow age-appropriate guidelines for strength and flexibility training. The ACSM also acknowledged the need for highly individualized exercise regimens for survivors of cancer given their differing fitness baselines, prognoses, sequelae of prior cancer treatment, sites of primary malignancy, and preferences. While pre-exercise testing was not deemed to be routinely necessary for the vast majority of patients, the guidelines did recommend general medical assessments for patients who were at-risk for neuropathy or musculoskeletal injury from cancer treatments, had known cardiac disease, or were at a high risk of fracture secondary to bone metastases prior to initiating structured exercise programs.

In addition, the American Cancer Society developed their own exercise guidelines with the goal of cancer prevention in 2012 [42]. These guidelines, in echoing the PAGA recommendations of 150 min per week of moderate-intensity or 75 min per week of vigorous-intensity exercise, stressed that any amount of physical activity was likely to have significant health benefits. A specific emphasis, in fact, was placed on reducing sedentary activities.

In 2018, HHS updated its PAGA guidelines with the “2018 Physical Activity Guidelines Advisory Committee Scientific Report” [43]. The Advisory Committee concurred with its previously issued 2008 exercise recommendations in terms of both duration and intensity. In addition, in its review of the impact of exercise on the prevention of cancer, they concluded that “strong evidence linked [the] highest versus lowest physical activity levels to reduced risks of bladder, breast, colon, endometrial, esophageal adenocarcinoma, renal, and gastric cancers” and “limited evidence suggests that greater amounts of physical activity are associated with a lower risk of head and neck cancer incidence”. The ACSM, in turn, concluded in a 2019 systematic review that “Levels of physical activity recommended in the 2018 Guidelines are associated with reduced risk and improved survival for several cancers”. However, it also noted that insufficient evidence existed to assess the impact of exercise on the risk of cancers of the head and neck [44].

While the above guidelines were developed independently, each has improved the general understanding of how patients with and survivors of cancer should approach physical activity and emphasized the importance of avoiding a sedentary lifestyle after a cancer diagnosis. Disease site-specific guidance remains an important consideration, as the tolerability, safety, and efficacy of specific exercise programs will be at least partially dictated by the sequelae of prior cancer-directed therapies. It will be important to keep this concept in mind when developing recommendations for patients with and survivors of cancers of the head and neck, as the comorbid conditions, functional status, and toxicity profiles of these patients may differ drastically from those that primarily informed the ACSM guidelines.

## 5. The Path Forward

There are multiple methods by which clinicians can promote participation in exercise by survivors of cancers of the head and neck. First, it is essential that these individuals undergo medical clearance by their entire healthcare team if they have any comorbidities that may affect the suitability of particular exercise regimens (for example, to screen for heart or lung disease, gait abnormalities, or bone disease). Additionally, consideration of the impact of sedative medications, such as opioid analgesics and muscle relaxants, which can increase the risk of falls, is vital when designing personalized exercise regimens. Second, patients should be specifically screened for psychological distress as a barrier to exercise using a standardized symptom assessment tool. Individuals who screen positive should be provided with specialized support from the clinical care team and referred to psychosocial specialists for further assessment and treatment as needed [45]. Third, encouraging different types of exercise activities, and designing strategies to incorporate physical activity into one’s activities of daily living, may help boost patient participation in exercise [46].

Fourth, strong social support systems have been shown to influence adherence to exercise regimens [47]. Therefore, patients should be encouraged to maintain personal support networks and to consider participation in formalized support groups. Fifth, patients should be thoroughly educated on the expected sequelae of definitive cancer treatment and the benefits of exercise, which may offer them the opportunity to take ownership of their health and to actively participate in mitigating the risk of long-term treatment complications.

Sixth, clinicians must shift their outlook. While the management of patients with cancers of the head and neck has focused primarily on definitive treatment in the past, an increasing focus must be placed on survivorship going forward. Maximizing post-treatment quality of life must become a core goal of caring for these patients, and medical care teams must reorient themselves to achieve this objective. Improving patient participation in regular exercise, with its myriad benefits, will go a long way towards achieving these ends.

Communication will prove key. As such, clinicians will need to hone their skills in order to encourage their patients to partake in exercise. In particular, providers should become proficient in motivational interviewing, a technique used to motivate patients to change their behavior [48]. Motivational interviewing has been shown to increase physical activity in patients with chronic health conditions [49]. Familiarity with the principles of self-determination theory, which seeks to understand the motivations behind individuals’ choices, will help complement these new clinical skills. Ample use of reflective listening and eliciting change talk, and learning how to align the importance of exercise with each patients’ long-term goals, may prove particularly effective in increasing activity levels [50,51].

In addition, clinicians should strongly consider the use of structured exercise programs and prehabilitation in this patient population. While some patients are able to initiate and manage their own exercise programs, involving exercise specialists, such as physical therapists and exercise physiologists, is strongly recommended, and the use of structured and personalized exercise regimens should be thoroughly considered. While there are limited data specifically exploring the role of structured exercise programs in patients with cancers of the head and neck, recent evidence suggests that they may be of particular benefit in this cohort. As noted above, in 2019, Samuel et al. published a trial of 148 patients with cancers of the head and neck undergoing treatment with definitive chemoradiation who were randomized to receive either an eleven-week structured exercise program or standard physical activity recommendations without supervision [24]. The exercise regimen in the experimental group consisted of aerobic and active resistance exercises completed five days per week for eleven weeks, and was initiated at the start of a seven-week hospitalization for treatment and continued for four additional weeks via a home-based program. Patients in the exercise group experienced significant improvements in terms of functional capacity (*p* < 0.001), quality of life (*p* < 0.0001), and preventing the worsening of fatigue (*p* < 0.001). These findings suggest that exercise-based programs in this patient population are well-tolerated and associated with significant improvements in patient-reported outcomes.

The idea of initiating exercise regimens prior to surgery in patients with cancer, termed “prehabilitation” or “prehab”, has also been an area of increasing interest in recent years. Surgery places enormous strain on the bodies of patients, and postoperative complications can be devastating, resulting in long hospital stays, permanent declines in functional status, and even death. Prehabilitation has been used to optimize the health of high-risk patients in the preoperative period in order to reduce the risk of these poor postoperative outcomes. While prehabilitation regimens vary, they may include consultations with counselors to curb the use of excess alcohol, tobacco, and illicit substances; nutritionists and dieticians to create individualized nutrition plans to reduce malnutrition and optimize weight; psychiatrists, psychologists, and/or therapists to optimize mental health and reduce stress; and physiotherapists and trainers to design and execute personalized exercise regimens [52,53].

Early data on the use of prehabilitation in patients with cancer have been encouraging. In 2019, a pooled analysis of three trials evaluating the role of prehabilitation therapy in patients with colorectal cancer noted an improvement in disease-free survival favoring the prehabilitation arm (73.4% vs. 50.9%; *p* = 0.04) [54]. Additionally, Michael et al. published a meta-analysis of five trials studying the role of prehabilitation exercise regimens in patients with cancer in 2021 [55]. A statistically significant improvement in the postoperative six-minute walk test distance was observed in the prehabilitation group as compared to the control group (mean difference: −58.0 m; 95% confidence interval: −92.8, −23.3 m). However, none of the included studies involved patients who were due to to undergo resection of a cancer of the head and neck. While the data supporting this practice specifically in patients with cancers of the head and neck are thus limited, the results published so far suggest that clinicians treating patients with cancers of the head and neck should at least consider the use of prehabilitation in appropriately selected patients. Further investigation of its benefit in the prospective setting should be strongly considered.

Based on the information summarized above, exercise prescriptions appear to be a promising avenue of future research with a number of potential benefits to patients with cancers of the head and neck. At this time, patients should be encouraged to follow the American Cancer Society guidelines of 150 min per week of moderate intensity exercise, such as walking for 30 min five times per week, or 75 min per week of vigorous exercise, such as running for 25 min three times per week [42]. Both aerobic and resistance exercises appear beneficial in this setting and a combined approach with the goal of improving both cardiovascular fitness and muscle strength simultaneously may be particularly beneficial. Additionally, patients should be screened for psychological distress, which may act as a barrier to exercise, and provided with treatment and appropriate referrals by the clinical care team prior to embarking on an exercise regimen. We would also recommend that providers begin discussing the benefits of exercise prior to the initiation of cancer-directed therapies and continue to encourage exercise beyond the completion of treatment. One method for completing this successfully could be to utilize motivational interviewing via telehealth platforms in order to engage patients more frequently and make interventions more accessible to patients who may live far away from cancer centers or have limited access to resources. Platforms that have been used include telephone calls, text messaging, and video communications. A recent review by Morrison et al. found that the utilization of telehealth platforms for exercise interventions was effective in reducing symptoms and associated with excellent treatment compliance [56]. These findings are particularly useful as we continue to navigate the COVID-19 pandemic.

## 6. Conclusions

As treatments for patients with cancers of the head and neck continue to improve, clinicians must increasingly emphasize their long-term health, especially in regard to minimizing the sequelae of definitive cancer treatment and maximizing quality of life. In recent years, the benefits of exercise in this patient population have increasingly been appreciated. While there are clearly many barriers to regular exercise in this cohort of patients, including psychological distress, medical comorbidities, and treatment toxicity, the benefits from exercise in survivors of cancers of the head and neck are potentially quite significant. Thus, while existing exercise guidelines lack specificity regarding best practices for this cohort of patients, clinicians should strive to encourage these individuals to engage in regular exercise.
